# Properties of Eco-Friendly Composites Based on Post-Consumer Recycled Resin Filled with Walnut Shell Powder

**DOI:** 10.3390/polym15224389

**Published:** 2023-11-12

**Authors:** Przemysław Pączkowski

**Affiliations:** Department of Polymer Chemistry, Institute of Chemical Sciences, Faculty of Chemistry, Maria Curie-Sklodowska University, Gliniana 33, 20-614 Lublin, Poland; przemyslaw.paczkowski@umcs.pl

**Keywords:** unsaturated polyester resin, agricultural waste, walnut shell, lignocellulosic biomass, composites, immersion test, accelerated aging, chemical resistance, mechanical properties, thermal properties

## Abstract

Increased demand for environmentally friendly materials resulted in a worldwide interest in manufacturing composite materials from agricultural wastes. Thus, this paper presents the results of research on the synthesis of eco-friendly composites and their properties. For their preparation, unsaturated polyester resin based on post-consumer recycled poly (ethylene terephthalate) was filled with walnut (*Júglans régia* L.) shell powder. After the filler incorporation, the deterioration of gloss and mechanical properties were observed. The flexural strength and modulus are significantly affected by the filler amount. Distilled water, 1% sodium hydroxide, toluene, and acetone were used as solvents in the chemical resistance test. Changes to the structure and properties of composites after 49 days of immersion in solvents were investigated. The immersion in water has no significant effect on the pure resin, but for its composites, the plasticizing effect of water was observed. The results show that all specimens show resistance toward toluene. In acetone, the resin and its composite shrink and fall into pieces, but the most destructive is an alkaline environment. After the immersion test, a huge increase in mass and a deterioration of gloss and mechanical properties were observed.

## 1. Introduction

Walnut (*Júglans régia* L.) is an important crop grown in all temperate regions of the world as edible nuts [1]. According to the FAO, worldwide walnut production in 2020 was approximately 3.3 million tons. China is the largest producer, with 1.1 million tons, followed by the USA, Iran, Turkey, Mexico, and Chile [2]. Since walnut shells constitute 67% of the total fruit weight [3], they create millions of tons of unused agricultural waste each year. As bio-waste, walnut shells have no economic value or industrial use and are usually thrown away, burned, or otherwise disposed of. Burning agricultural waste causes serious environmental problems.

The main component of these waste materials is lignocellulosic material. The lignocellulosic material contained in walnut shells has many chemical advantages, such as a large number of reactive functional groups, high carbon content, compatibility with a variety of industrial chemicals, and good stability. It also has good mechanical properties resulting from the presence of aromatic rings, and good rheological and viscoelastic properties, which indicate the possibility of its use as a potential candidate as a reinforcing material in polymer composites. The chemical structure of walnut shells consists mainly of cellulose (23.9%), hemicellulose (22.4%), lignin (50.3%), and ash (3.4%) [4]. In addition to the main ingredients, the shells also contain extractive substances such as tannins, pectins, fats, waxes, resins, essential oils, and volatile substances [5].

Walnut shells as a reinforcing filler are especially intended for composites containing thermoplastic polymers, for external applications requiring high dimensional stability. These properties are influenced by the low content of hygroscopic materials (cellulose and hemicellulose) and the higher content of hydrophobic materials (lignin and extracts) [6]. However, it should be noted that too high a content of extractive substances combined with poor wettability of the walnut shell may lead to a weaker bond between the filler particles and weakening of the bonding forces in the final product.

Over recent decades, research has been carried out on the use of walnut shells as a reinforcing material for the manufacture of composite made of high-density polyethylene (HDPE), low-density polyethylene (LDPE), polylactide and poly (ε-caprolactone), thermoplastic starch, polypropylene (PP), ethylene propylene diene monomer rubber (EPDM) and natural rubber (NR) [7,8,9,10,11,12,13,14,15].

Unsaturated polyester resins (UPRs) are an important class of high-performance engineering polymers used in many applications primarily in compression molding, injection molding, resin transfer molding, pultrusion, filament/fiber winding, and hand lay-up process. These thermosetting matrixes have been used for the fabrication of uncountable composite materials for decades due to their availability at a competitive price worldwide [16], which is why 85% of reinforced polymer products such as boat, car, and airplane parts or chairs were made from them [17]. Due to sustained interest, the global unsaturated polyester resin market is expected to have a compound annual growth rate (CAGR) of 7.1% over the forecast period of 2023–2030 [18]. The UPRs are prepared by dissolving the starting unsaturated polyesters in vinyl monomers, mainly styrene [19].

Poly (ethylene terephthalate) is one of the largest components of post-consumer plas-tic waste ending up in landfills, the main source of which are soft-drink bottles. For this reason, there is a great interest in its reprocessing and use, saving the natural environ-ment. PET is recycled through glycolysis with diethylene glycol. The obtained ethylene terephthalate and its oligomers are polycondensed with maleic anhydride and phthalic acid. From the obtained recyclate, unsaturated polyester resins as thermosetting matrixes can be produced, thus creating a valuable product [20,21,22,23,24].

The aim of this paper was to investigate the effect of walnut (*Júglans régia* L.) shell powder incorporation into the unsaturated polyester resins on the chemical resistant, physical, thermal, mechanical, optical, morphological, or structural properties. The results from the immersion test and accelerated aging of composites were discussed. The motivation for these studies was to learn about the performance properties of composites subjected to unfavorable environmental conditions, such as water, or long-term exposure to the outdoors. This paper is a continuation of the research carried out by the author on the possibility of using alternative raw materials for the production of polyester composites.

## 2. Materials and Methods

### 2.1. Chemicals

The solution of styrene with an unsaturated polyester resin based on the post-consumer recycled poly (ethylene terephthalate) (PET) soft-drink bottles, Estromal 14PB-06 NZ (a bluish-green liquid, non-volatile content 61.2 wt.%, viscosity at 23 °C 356 mPas, acidic value 13.4 mg KOH g^−1^, reactivity factor 1.53, gel time 14 min, and maximum copolymerization temperature 164.8 °C) was provided by LERG (Pustków, Poland). Methyl ethyl ketone peroxide (MEKP, Luperox DHD-9) as an initiator was provided by Sigma-Aldrich (St. Louis, MO, USA). A 4% solution of polymeric cobalt as an accelerator was synthesized in the Department of Polymer Chemistry, Maria Curie-Sklodowska University (Lublin, Poland).

### 2.2. Filler Preparation

Chilean walnut (*Júglans régia* L.)-dried shells were ground in an analytical mill A11 basic from IKA Werke GmbH & Co. KG (Staufen, Germany). The obtained fractions were separated using a vibratory sieve shaker. For composite preparation, walnut shell powder (WSP) from the 0.25–0.10 mm fraction was taken.

### 2.3. Walnut Shell Powder Composites

The polymeric composites were prepared by mixing the unsaturated polyester resin (UPR) with different weight percentages of walnut shell powder (2, 5, 7, and 10). Based on the resin content, 1.1 wt.% MEKP as an initiator and 0.25 wt.% of 4% polymeric cobalt solution as an accelerator were added.

The mixture was well stirred until homogeneity was reached, and degassed by a vacuum kit from VacuumChambers.eu (Białystok, Poland), and then poured into the cuboid-shaped mold. The prepared composites were allowed to be cured at room temperature for 24 h and then post-cured in the oven at 80 °C for 10 h.

### 2.4. Composite Specimens

**Figure 1:**
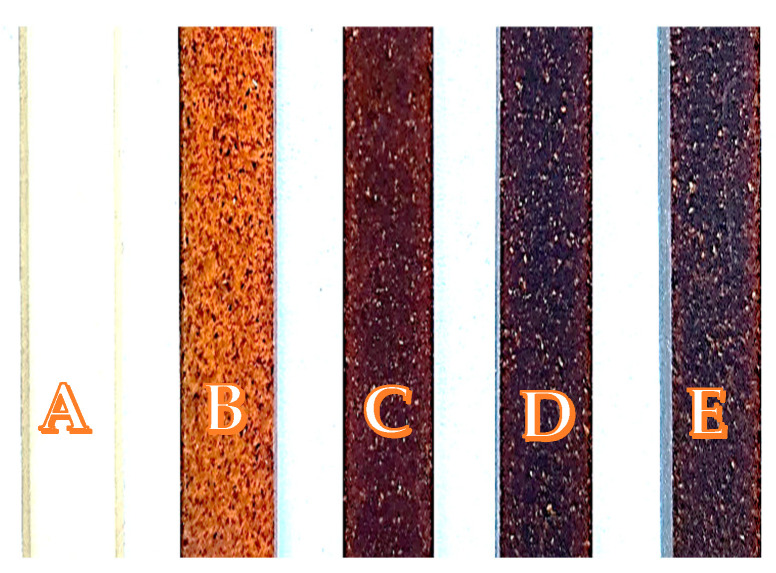
UPR samples: (**A**)—pure UPR; (**B**)—UPR + W2; (**C**)—UPR + W5; (**D**)—UPR + W7; (**E**)—UPR + W10.

Composite samples for resistance tests were cut in the form of cuboids with dimensions of 80 mm × 10 mm × 4 mm (thickness) using a CNC milling machine MFG 8037P from Ergwind (Gdańsk). The specimens were subjected to immersion testing (in various solvents), accelerated aging (in a chamber), or exposure to microwave radiation (reactor). A sample that was not used in degradation studies was taken as a reference (Figure 1).

## 3. Research Methods and Characterizations

### 3.1. Chemical Resistance

The behavior of UPR/WSP composites in the presence of liquids was determined. The test procedure was in accordance with the standard EN ISO 175:2010 [25]. The 80 mm × 10 mm × 4 mm specimens were immersed separately in an airtight container with 50 mL of the test liquid and placed in a dark place at room temperature (23 °C ± 2 °C). Distilled water, 1% aq. NaOH solution, acetone, and toluene were used as test liquids. After immersion for a specific 1-week interval, the samples were periodically withdrawn from liquids, rinsed with distilled water, and gently wiped off. Changes in mass, dimensions, and color or other appearance attributes were determined.

The mass change Δ*m* is expressed by Equation (1) [25]:

(1) $$∆m=\frac{m_{i}-m_{0}}{m_{0}}\times 100$$

where *m*_0_ is the initial mass of the specimen and *m_i_* is the mass of the immersed specimen.

The relationship between the time of immersion and the mass change in UPR-based materials is presented in the graphs.

### 3.2. Gloss Measurement and Optical Properties

Gloss measurement was carried out using the triple-angle gloss meter, Zehntner ZGM 1110 from Zehntner GmbH Testing Instruments (Sissach, Switzerland). The device allows for simultaneous display value at 20°, 60°, and 85°, corresponding from high gloss to matte surface. The gloss value of a highly polished black glass standard was 86.8 GU (20°), 93.4 GU (60°), and 99.7 GU (85°). These determinations were made according to the standard ASTM D2457 [26] where the final result was the arithmetic averaging of ten measurements before and after the degradation.

The UV-visible spectra of the specimens were obtained using a UV-2550 spectrophotometer from Shimadzu (Kyoto, Japan) at room temperature (23 °C ± 2 °C) in the wavelength range from 190.0 to 1000.0 nm. The transmittance and absorbance at 0.5 nm sampling intervals and a light source change wavelength of 295 nm were measured.

### 3.3. Thermal Stabilities

Thermogravimetry (TG) and differential scanning calorimetry (DSC) were used to study the thermal stability of walnut shell powder and UPR composites. TG/DTG/DSC data were collected using a Netzsch STA 449 F1 Jupiter (Selb, Germany) over a temperature range of 30 to 1000 °C at a heating rate of 10 °C min^−1^ in an oxidizing atmosphere (air). The tests were carried out in accordance with the EN ISO 11358-1:2014 standard [27].

### 3.4. Hardness and Mechanical Properties

The effect of walnut shell powder on the hardness of the UPR composites was determined using a Barcol impressor hardness tester GYZJ 934-1 from Barber-Colman Company (Loves Park, IL, USA). The procedure followed the ASTM D2583 at standard temperature (23 °C ± 2 °C) [28]. Finally, the arithmetic averaging of ten individual measurements of resin casting was taken.

The mechanical properties of the UPR/WSP specimens were determined on the Zwick/Roell Z010 universal testing machine from Zwick GmbH Co. (Ulm, Germany) at room temperature with a load capacity of 10 kN. Determination of properties based on the three-point bending flexural test with speed was 5 mm/min, where the samples of 80 mm × 10 mm × 4 mm diameter were used with a span of 64 mm between the supports. The flexural strain at break ($\varepsilon_{f}$), flexural modulus ($E_{f}$), and flexural strength ($\sigma_{f}$) were determined. The measurements were made according to the EN ISO 178:2019 standard [29].

### 3.5. Accelerated Aging

The accelerated aging test was performed using the Atlas Xenotest Alpha+ simulator (Chicago, IL, USA). The source of irradiation is a xenon lamp emitting radiation similar to natural sunlight with a power of 60 W m^−2^. The chamber temperature was 38 °C, the black reference temperature (65 °C), the relative humidity was 50%. The dry exposure time was 200 h. The above parameters reflected the weather conditions to which the materials may be exposed. Each sample was exposed to a dose rate (NTM) of approximately 43,200 kJ m^−2^. The test was carried out in accordance with the EN ISO 4892-2:2013 standard [30].

### 3.6. Microwave Treatment

The microwave irradiation of the UPR/WSP composites was performed using a MAS-II Plus Microwave Synthesis Workstation from SINEO Microwave Chemistry Technology Co., Ltd. (Shanghai, China). The samples were exposed to microwaves with a power of 1000 W until the temperature reached 150 °C for 20 min.

The relationship between the time of irradiation and the mass change in UPR composites was presented.

### 3.7. Elemental Analysis and FT-IR Spectroscopy

Elemental analysis of the walnut shell powder was carried out using a CHNS-analyzer EuroEA3000 from EuroVector (Pavia, Italy). The device allows for simultaneous determination of the percentage of carbon (%*C*), hydrogen (%*H*), nitrogen (%*N*), and sulfur (%*S*) in the sample. The oxygen content (%*O*) is determined using Equation (2) [22]:

(2) %O=100%−(%C+%H+%N+%S)

Finally, the arithmetic averaging of two individual measurements was taken.

Fourier transform infrared (FT-IR) spectroscopy was used to characterize the functional groups present in the walnut shells. The spectra were measured on Bruker TENSOR 27 spectrometer (Ettlingen, Germany) with a resolution of 4 cm^−1^ in the range from 600 to 4000 cm^−1^ using 32 scans per sample. Analysis preceded by background spectrum measurement.

### 3.8. Morphology and Microstructure

The micrographs determining the morphology, structure, and texture of powder from walnut shells were taken using the FEI QUANTA 3D FEG high-resolution scanning electron microscope (Hillsboro, OR, USA) at an acceleration voltage of 5 kV. To avoid electrostatic charging during the examination, the sample was covered with a thin layer of Pd/Au.

## 4. Results and Discussion

### 4.1. Characterization of Eco Filler and Post-Consumer Resin

The discussion of the results began with the agricultural waste filler characteristics. According to the CHNS elemental analysis data, the walnut shell powder is composed of carbon (45.98%), hydrogen (6.10%), and nitrogen (0.85%). It can be assumed that the remaining value of 47.07% is oxygen (Equation (2)). Generally, those mentioned elements build cellulose, hemicellulose, and lignin, the main components of the walnut shell [4].

This agricultural waste filler offers multiple functional groups, such as carboxyl, carbonyl, hydroxyl, and amine in characteristic chemical structures. As expected, the structural characterization of this lignocellulosic material by FT-IR spectrum is extremely complicated because it is caused by many different groups involved in the cellulose, hemicellulose, and lignin molecules. The assignment of the bands is consistent with that reported in many articles on lignocellulosic material [31,32,33].

**Figure 2:**
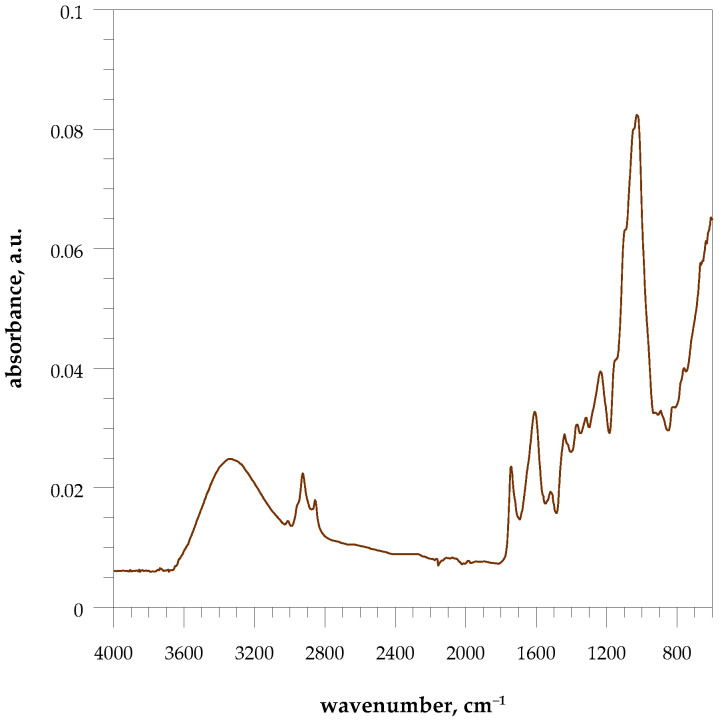
FT-IR spectra of walnut shell powder (WSP).

The FTIR spectrum, Figure 2, was used to verify the presence of the organic groups, mainly due to lignin, cellulose, and hemicelluloses, from walnut shells.

The filler sample can be verified by a broad band at 3343 cm^−1^, which is characteristic of stretching vibrations for OH functional groups of alcohols, phenols, carboxylic acids, cellulose, hemicellulose, lignin, and adsorbed water. In addition, bands at 3010 cm^−1^, 2926, and 2855 cm^−1^, can be assigned to the C–H stretching of aromatic groups, and C–H vibration of aliphatic groups methyl and methylene, respectively. Additionally, the band at 3010 cm^−1^ can be related to C=CH stretching vibration peak in fatty acids.

Absorption bands at 1743, 1608, 1518, and 1439 cm^−1^ are associated with functional groups present in lignin. The band at 1743 cm^−1^ can be also related to the carbonyls (C=O) group of acetyl and esters in hemicellulose and to the ester linkages of the hydroxycinnamic acids’ carboxylic groups that bind lignin and hemicellulose. The band at 1608 cm^−1^ is related to C=O stretching vibrations from the aromatics skeletal (hemicellulose and lignin). Absorption bands at 1608, 1518, and 1439 cm^−1^ super-imposed on C=C stretching vibrations in the aromatic ring.

Some bands are characteristics of lignin like the 1234 and 1439 cm^−1^, which may be due to the C–O stretching of guaiacyl rings and CH methyl groups, respectively.

Absorption bands at 1153, 1093, 1047, and 1027 cm^−1^ are associated with the C–O–C stretching vibration (cellulose, hemicellulose, anhydrides, ethers, esters, phenols, carboxylic acids and derivatives), aromatic C-H in-plane deformation (lignin), C–OH stretching and C–O deformation (cellulose, hemicellulose, lignin, polysaccharides), and C–O stretching, aromatic C–H in-plane deformation (cellulose, lignin), respectively.

Absorption bands at 894, and 824 cm^−1^ are associated with stretching vibrations of C–O–C glycosidic linkage and to CH_n_ aliphatic or aromatic bonds, i.e., CH deformation of glucose ring in cellulose and hemicellulose.

**Figure 3:**
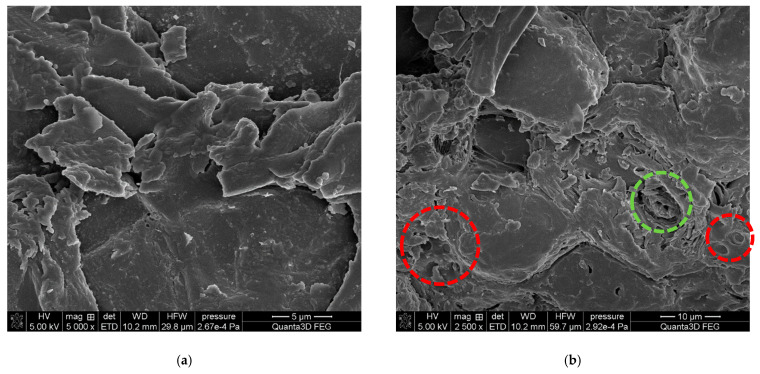

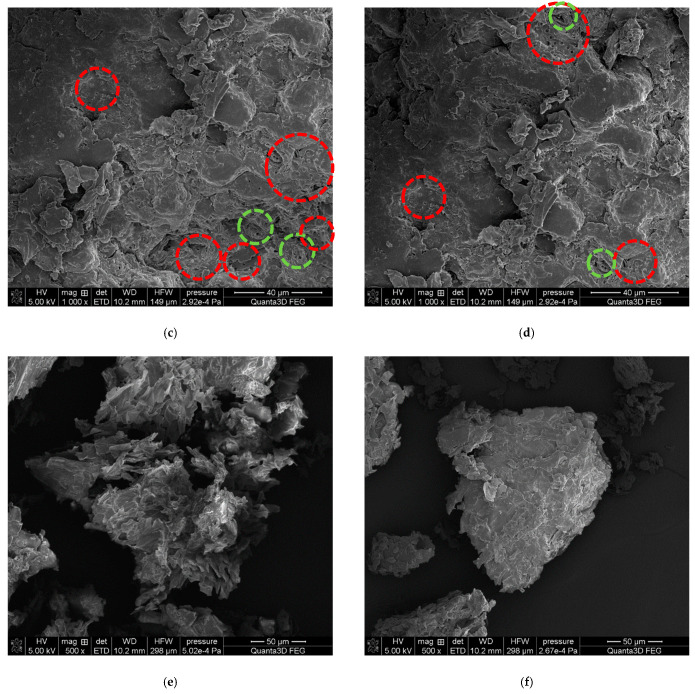
SEM micrographs of the ground walnut shells at different magnifications.

The morphological analysis of the WSP was performed using scanning electron microscopy at different magnifications (Figure 3). The micrographs of ground walnut shells showed fine sheet- or plate-like structures, creating aggregates with sharp edges (Figure 3a,e,f). The simple pitted cell walls with caves or pores (marked with red circles) and the rolled sheets architecture (green circles) were also observed (Figure 3b–d).

The unsaturated polyester resin was synthesized from the post-consumer recycled poly (ethylene terephthalate). Bis (2-hydroxyethyl) terephthalate obtained in the glycolysis process of PET wastes was then condensed with maleic anhydride and phthalic acid. To prepare the UPR, unsaturated polyester was mixed with styrene. The structural characterization of cured polyester has been described earlier [34].

### 4.2. Thermal Properties of Composites

To study the stability and the stages of degradation of the filler and polymeric matrix during heating in the oxidative atmosphere, thermogravimetry, and differential scanning calorimetry analyses were taken. The obtained numerical data for the walnut shell flour and UPR composites were summarized in Table 1 while their TG-DTG thermal decomposition curves are presented in Figure S1 (Supplementary Materials).

The effect of the temperature on the biocomposite can be connected with the resin and fibers thermolysis mechanism. The first one is the scission of chemical bonds of the resin polymeric matrix and the second one is the thermal degradation of fibers.

From the TG and DTG curves, it can be seen that the cured pure unsaturated polyester has two main thermal degradation steps. A random chain cracking with a decomposition maximum at 388 and 520 °C occurred (Figure S1a). From a thermal point of view, the weakest points of the polyester material are two different types of ester groups. The first of them in aliphatic chains comes from maleic anhydride, while the second ones stabilized by their proximity to aromatic rings, decompose at a temperature of 388 °C [35]. Due to the presence of oxygen, degradation at a temperature of 520 °C corresponds to the combustion process [36].

According to the TG and DTG curves of the walnut shell flour, the initial weight loss starts at quite low temperatures. Despite the fact that the filler was dried, about 8.20% of some moisture and volatile compounds still evaporated to a temperature of about 120 °C, and a similar observation for typically lignocellulosic filler was noticed in previous work [34,37]. It can be assumed that the degradation of the main components of the WSF takes place in the other stages. According to Azwa et al. [38], the thermal degradation of natural fibers is a multi-stage process that begins above 100–120 °C by the evaporation of moisture. The TG curve of shell powder shows a significant loss (86.31%) in the wide temperature range of 190–500 °C. Few peaks on the DTG curve at 228, 277, and 404 °C could be correlated with the dehydration, decomposition, depolymerization, and degradation of the hemicellulose, cellulose, and lignin (Figure S1b) [39].

For the composites, the more complex process of thermo-oxidative degradation is based not only on the thermal decomposition random chain scission and unzipping depolymerization reactions in the polymer matrix but also in the filler components like cellulose, hemicellulose, or lignin (Figure S1c–f).

In the case of UPR-based materials with walnut shell powder, two stages of degradation analogous to the pure UPR were also observed. For the composites, weight change decreased in the first decomposition area from 83.30 to 76.80%, and in the second, it increased from 14.83 to 19.54%. The filler incorporation had also an effect on the residual mass even at 1000 °C. For the UPR + W2, it was 0.25%, and it increased to 2.27% for the UPR + W10 while for the pure resin, no ash was formed.

An increase in filler amount in the composite resulted in a value change in the characteristic temperatures. The temperatures at 5%, 10%, and 50% mass loss of the pure resin were 319, 340, and 390 °C, respectively and gradually decreased to 292, 330, and 385 °C as the walnut shell powder content increased. The values of maximum decomposition temperature with the filler incorporation increased a bit in the first decomposition area from 388 to 391 °C, while a significant loss in the second decomposition area from 520 to 471 °C was noticed.

Calorimetric analysis using differential scanning calorimetry allows us to understand a wide range of physical changes that a material undergoes under exposed to temperature. DSC curves are presented in Figure S2 (Supplementary Materials).

The thermal transformations observed at the DSC curve for walnut shells include a broad peak from the beginning of the measurement to 120 °C with a maximum at 74.3 °C assigned to the endothermic evaporation of moisture, and a broad peak from 200 to 500 °C with maxima at 287.8, 410.3, and 430.8 °C related to exothermic decomposition processes.

For the pure UPR, only a broad exothermic peak in the range 300–550 °C with maxima at 395.0, 493.4, and 521.8 °C was observed. The situation is almost analogous for all UPR/WSP composites, where the shift of the other degradation processes to higher temperatures was noticed.

### 4.3. Mechanical Properties of Composites

An increase in void content due to the filler incorporation indicates poor-quality composites and changes in mechanical properties. Based on the carried out experiments it can be concluded that the void content percentage increases with an increase in the concentration [40].

**Figure 4:**
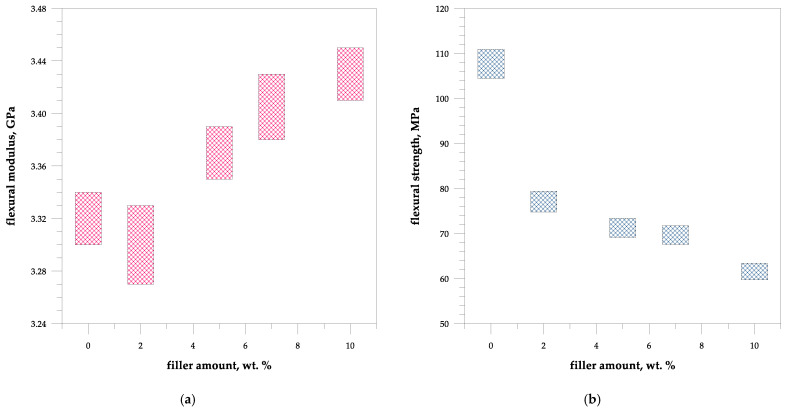
Mechanical data of the UPR/WSP composites: (**a**) Flexural modulus, (**b**) Flexural strength.

The incorporation of walnut shell powder into the unsaturated polyester resin caused first a slight reduction of flexural modulus value due to the disruption of the matrix polymeric network system and then its increase to 3.43 GPa (Figure 4). Walnut shell flour is characterized by rigidity, which in turn increases the stiffness and reduces the elongation of the polymeric material. More brittle behavior of the reinforced unsaturated polyester resin was also confirmed by hardness determination where for the pure UPR and composites, the value increased from 43 to 52 HBa.

However, the walnut shell particles decrease flexural strength and strain at the break of polyester composites from 107.69 to 61.55 MPa, and 3.46–1.91%, respectively due to porosity, poor adhesion and poor interfacial interaction between the polyester resin and the walnut shell particles The reduction of the strength indicates that filler debonded from the polyester matrix before the material experienced profound plastic deformation. The strain at break decreases with increasing filler content and this is a common observation with almost all filled polymers. This phenomenon is caused due to the decreased deformability of rigid interphase between the filler and the matrix.

### 4.4. Influence of Microwaves on Composites

**Figure 5:**
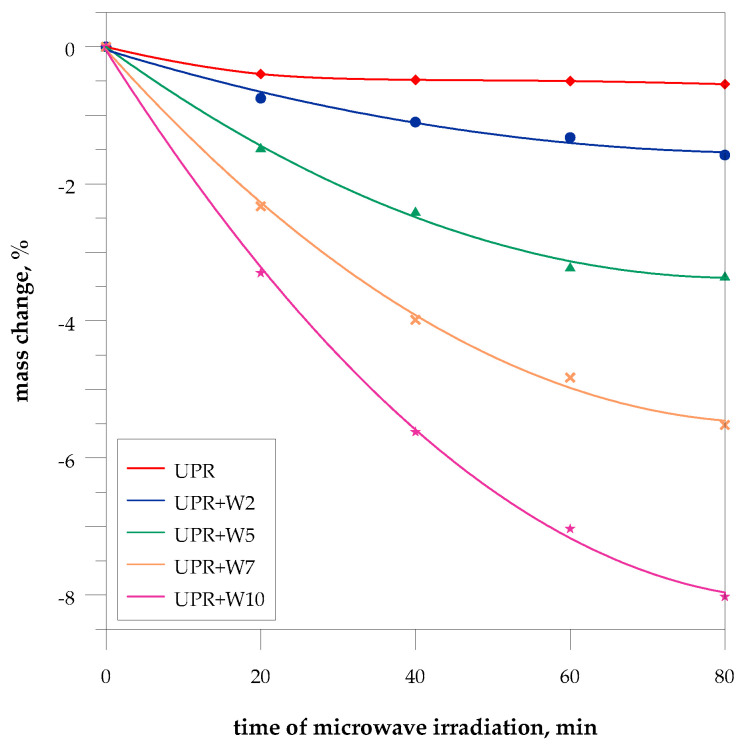
Mass change in the UPR/WSP composites during microwave irradiation.

The tested samples were placed in a microwave reactor on ceramic pedestals and exposed to microwave power of 1000 W. The change in the mass of the samples was examined after 20, 40, 60, and 80 min. irradiation. The obtained results are presented in Figure 5. These data show that microwaves cause a very small mass loss of pure UPR. However, in the case of composites, it is much larger.

As a result of microwave exposure, the pure polyester matrix had a flat, smooth surface, but significant yellowing and even browning of the sample was observed. In the literature, it can be found that the resin-based materials undergo a secondary curing reaction [41,42,43]. The observed relatively stable mass after 20 min can suggest that the cross-linking or moisture evaporation processes have already been completed. The microwave-assisted post-curing of the polyester matrix was confirmed by a slight increase in the hardness value from 43 to 44 HBa suggesting almost complete bond conversion of the comonomers after thermal post-curing.

In the case of composites, the microwave-assisted degradation occurred faster due to the destruction of the filler components. A significant change was noticed in the appearance of the sample surface, where cracking and resembling a mosaic structure or patterns were observed.

According to the TG-DTG data (Figure S1b), the filler contains over 8% moisture and volatile substances (organic fats, waxes, resins, essential oils, and volatiles), which evaporate during microwave treatment.

The microwaves may initiate reactions between the filler components and also them with the polyester matrix, resulting in the formation of new bonds, and volatile compounds and consequently, their further degradation.

### 4.5. Influence of Solvents on Composites

Generally, solvent diffusion in biocomposites leads to physical and chemical degradation. Physical aging of materials involves plasticization and swelling of the polymer matrix [44]. The latter may lead to degradation of the contact surface, such as filler debonding and delamination [45]. Chemical degradation of composites mainly involves hydrolysis of the polyester matrix, cracking of the matrix, and degradation of the matrix-filler interface [46].

**Figure 6:**
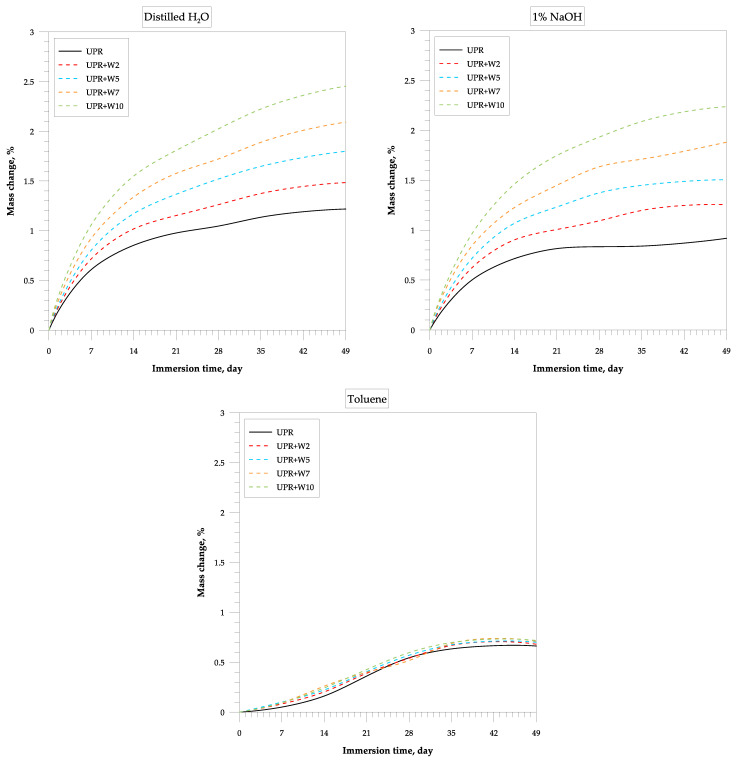
Effect of the chemical resistance of the UPR/WSP composites immersed in the distilled water, alkaline solution, and toluene.

**Figure 7:**
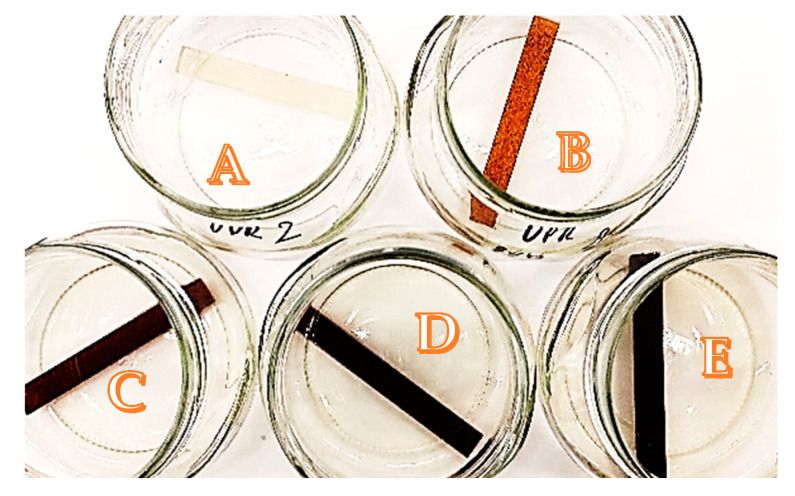
UPR samples during immersion test in alkaline solution: (**A**)—pure UPR; (**B**)—UPR + W2; (**C**)—UPR + W5; (**D**)—UPR + W7; (**E**)—UPR + W10.

Figure 6 and Figure 7 show the changes occurring in resins and their composites with shell powder during the immersion test. Many changes were observed not only in the materials themselves but also in some of the liquid chemicals contained in which they were immersed. For example, severe shrinkage was observed in acetone, which caused the polymer matrix to crack after just one day.

The same phenomenon was observed for the UPR composites with walnut shell powder. However, with the addition of the filler, such a strong shrinkage was not observed, but the process of partial delamination and exfoliation took place.

Figure 6 shows the relationships between the mass of the samples and their immersion time. It shows exemplary curves obtained in distilled water, alkali (1% NaOH aq. solution), and toluene during the 49-day test. The order of the curves is always the same where the smallest mass changes after the immersion were shown by the pure cured polyester resin. The UPR composites with walnut shell powder exhibited the largest weight gain. Generally, all composite samples are characterized by similar behavior in the aqueous solutions where the greatest weight gain can be observed in distilled water.

The kinetics of water diffusion were found to be the same for composites immersed in distilled water and alkaline solution at room temperature, but weight gain was higher for distilled water. The explanation for this phenomenon is that sodium-based crystals can act as a barrier by blocking the paths through which water diffuses into the material polymeric matrix [47].

As presented in Figure 7, for the UPR/WSP composites during the immersion test, many changes were observed not only in the materials themselves but also in the liquid chemicals in which the samples were immersed. Beg and Pickering [48] reported the leaching of lignin and water-soluble products from composite samples during aging. A similar observation was also noticed in previous works [34,37,49].

Color changes in liquid chemicals were observed for alkaline (Figure 7B–E) where yellowish or orange-yellowish solutions included leaching of the lignin from the biofiller. As was expected, more intense color was corresponding with the filler amount in the composites, especially as in Figure 7D,E.

### 4.6. Surface Gloss of Composites

**Figure 8:**
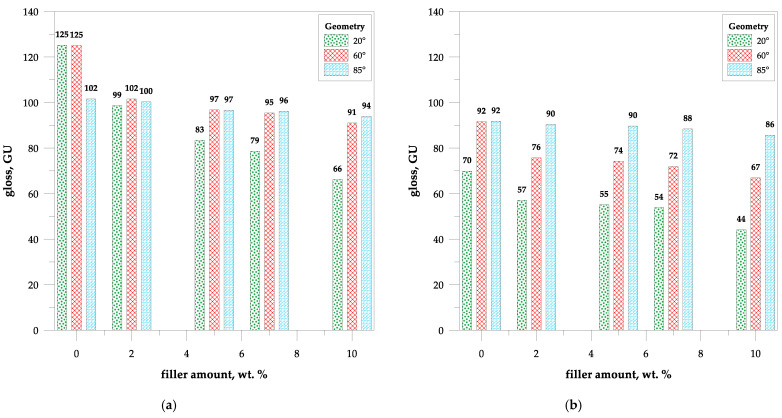
Gloss data of UPR/WSP composites: (**a**) before and (**b**) after accelerated aging.

Gloss is known to be an important parameter characterizing the surface of the material. The influence of the immersion test on the gloss of the samples is presented in Figure 8. The 60° geometry can be used for all materials; however, for very high gloss, the 20° geometry measurement method is recommended.

According to gloss data, one can see that the parameter decreased with the addition of the filler. All samples can be considered high-gloss materials, as their 60° geometry values were greater than 70 GU. The parameter ranged from 125 to 91 GU for the pure UPR and 10 wt.% composite, respectively (Figure 8a).

The pure polyester matrix loses gloss due to accelerated aging under UV light (Figure 8b). One of the materials, the one with the highest filler loading, is no longer highly glossy (67 GU). However, with the increase in the filler content in composites, the gloss after aging changes less compared to the references from −26.4 to −23.7%. This suggests that lignin, cellulose, and hemicellulose present in the filler absorb UV light and prevent the photochemical degradation of the polymeric matrix. On the other hand, the presence of chromophores and aromatic rings makes the filler susceptible to decomposition in photooxidation reactions [50].

### 4.7. Optical Properties of Composites

**Figure 9:**
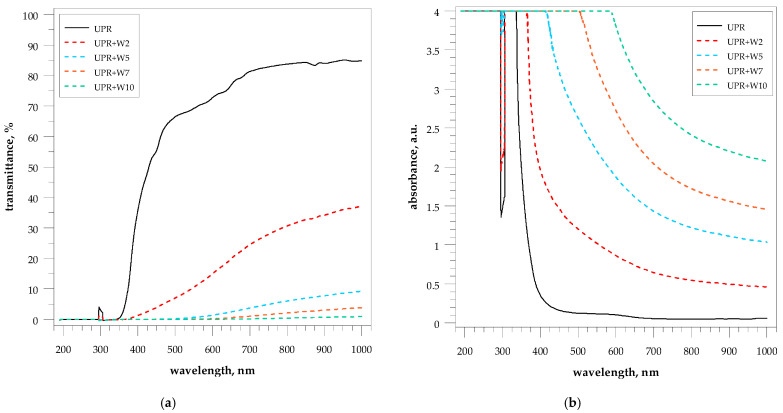
UV-VIS-NIR transmittance (**a**) and absorbance (**b**) spectra of the UPR/WSP composites.

The optical properties of the pure UPR and composites with walnut shell powder were determined by the UV-visual spectroscopy measuring transmittance and absorbance in the wavelength range from 190 to 1000 nm (Figure 9, Table 2). The thickness of the test samples was ~4 mm.

The pure UPR sample was characterized by the highest transmittance value in the wavelength range from about 320 to 1000 nm among all samples (Figure 9a).

Opacity is known to be a function of the bending of the optical path of white light in such a way that its path is reversed and returns to the viewer’s eye. The addition of walnut shell particles has a significant effect on the transparency of the UPR.

The interaction of light with the filler is very strongly dependent not only on the size of the filler particles but above all also on its amount. Therefore, a significant decrease in transmittance was observed already for a sample containing only 2 wt.% of filler. The next significant decrease is observed for material containing 5 wt.% of walnut shell powder. Above such an amount of filler, it is difficult to talk about any beam penetration through the materials where the transmittance was less than 5% at 950 nm.

Pure UPR possesses e.g., aromatic groups with strong absorption in ultraviolet light. The spectrum of this sample was also characterized by almost no absorbance in the visible range (Figure 9b).

For the walnut shell composites, the bathochromic shift of the absorbance spectrum was observed in the entire measurement range. This can be explained by the appearance of new chromophores derived from the walnut filler. The spectra also show the dependence of the absorption and the amount of filler, where absorption increases with the increase in the amount of walnut shell powder.

## 5. Conclusions

Walnut shell powder is inexpensive, readily available, and tends to be processed with various types of polymer matrixes as agricultural waste material. Composites based on unsaturated polyester resin with different amounts of walnut shell flour were obtained and investigated given their structural, thermal, mechanical, optical, and chemical resistant properties. The results show that the commercially available resin based on post-consumer recycled PET can be used for the preparation of eco-friendly biocomposites with walnut shells up to 10%.

Based on the research, it was found that the filler is a material reinforcement that partially absorbs UV radiation and thus extends the life of the polymer matrix. Walnut, due to its lignocellulosic origin, is a material that is poorly resistant to high temperatures, causing deterioration of the thermal properties of the composite, and ash is formed during the thermal oxidation process. When using microwaves on a polymeric material containing waste material, its destruction occurs first, while the polyester matrix has hardened. Along with the exposure time, the evaporation of compounds from the filler and its charring destroyed the material. The presence of a filler affects the ability to absorb certain solvents. The changes in the properties of the eco-friendly composites after immersion are dependent on the chemical nature of the solvent in which they were immersed. For these materials, the delignification process in an alkaline environment could be observed.

Developing a new material using two waste materials such as walnut shells and polyester resin based on post-consumer recycled PET results in environmental benefits. Due to their low weight, low cost, and good resistance to many external factors, these composites are a potential alternative material that can be used in the household or construction as a material mimic of wooden boards in siding panels.

## Figures and Tables

**Table 1:** Thermogravimetric analysis data for the UPR composites with walnut shell powder.

*Sample*	$T_{5\%}$ (°C) ^1^	$T_{10\%}$ (°C) ^2^	$T_{50\%}$ (°C) ^3^	$T_{max}$ (°C) ^4^	MC (%) ^5^	RM (%) ^6^
Walnut Shell Powder (WSP)	75.9	197.5	290.8	66.7; 227.9 277.1; 404.0; 662.6	−8.20; −10.48; −53.73; −22.10; −2.15	3.34
pure UPR	319.4	339.8	390.3	387.9; 520.4	−83.30; −14.83	---
UPR + W2	312.5	340.3	388.2	388.1; 494.3	−82.81; −15.48	0.25
UPR + W5	308.3	337.6	387.7	390.1; 489.1	−80.27; −16.57	1.36
UPR + W7	300.7	334.3	386.6	390.6; 473.2	−77.73; −18.15	1.81
UPR + W10	292.2	329.8	385.4	391.1; 470.8	−76.80; −19.54	2.27

^1^ Temperature of 5% mass loss; ^2^ Temperature of 10% mass loss; ^3^ Temperature of 50% mass loss; ^4^ Maximum decomposition temperature; ^5^ Mass Change; ^6^ Residual Mass at 1000 °C.

**Table 2:** Optical properties of the UPR/WSP composites with walnut shell powder.

Sample	Optical Properties at Wavelength:													
	350 nm		450 nm		550 nm		650 nm		750 nm		850 nm		950 nm	
	T ^1^	A ^2^	T	A	T	A	T	A	T	A	T	A	T	A
pure UPR	0.446	2.398	55.327	0.258	69.177	0.161	76.846	0.116	82.782	0.083	84.245	0.076	85.078	0.072
UPR + W2	---	4.000	3.828	1.468	10.521	1.019	19.865	0.738	27.855	0.588	32.706	0.517	36.015	0.475
UPR + W5	---	4.000	0.058	3.217	0.627	2.200	2.434	1.613	4.884	1.311	6.950	1.158	8.569	1.068
UPR + W7	---	4.000	---	4.000	0.063	3.264	0.543	2.318	1.549	1.854	2.571	1.629	3.444	1.500
UPR + W10	---	4.000	---	4.000	---	4.000	0.064	3.211	0.273	2.587	0.545	2.289	0.790	2.128

^1^ Transmittance, %; ^2^ Absorbance, au.

## Data Availability

No new data were created or analyzed in this study. Data sharing is not applicable to this article.

## Supplementary Materials

The following supporting information can be downloaded at: https://www.mdpi.com/article/10.3390/polym15224389/s1, Figure S1: Thermal decomposition of the UPR/WSP composites; Figure S2: DSC curves of the UPR/WSP composites.
