# Supplementary material for: Properties of Eco-Friendly Composites Based on Post-Consumer Recycled Resin Filled with Walnut Shell Powder
Source: Polymers (Basel). 2023 Nov 12;15(22):4389. doi: 10.3390/polym15224389 (PMC10674546; doi:10.3390/polym15224389)
Supplement: Supplementary file 1 [file polymers-15-04389-s001.zip › polymers-2599000-supplementary.pdf]

Supplementary Materials

Properties of Eco-Friendly Composites Based on Post-Consumer Recycled Resin Filled with Walnut Shell Powder

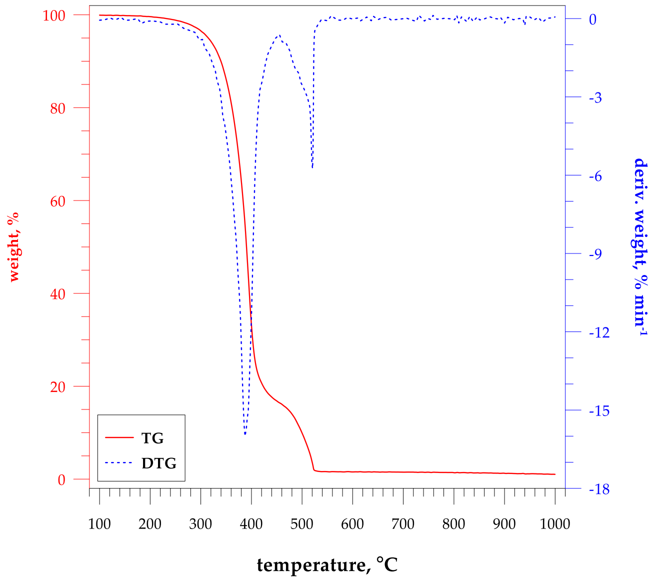

(a)

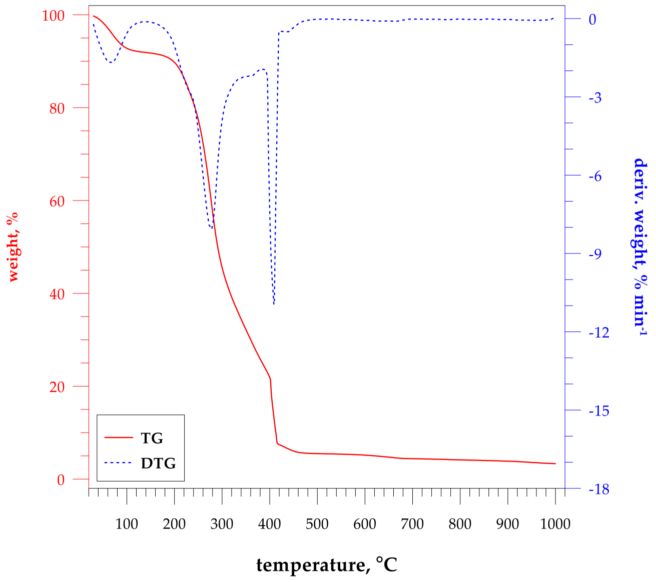

(b)

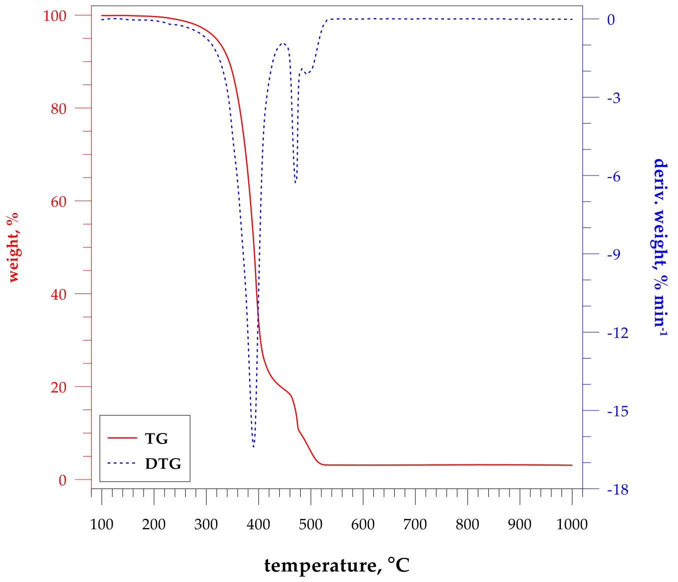

(c)

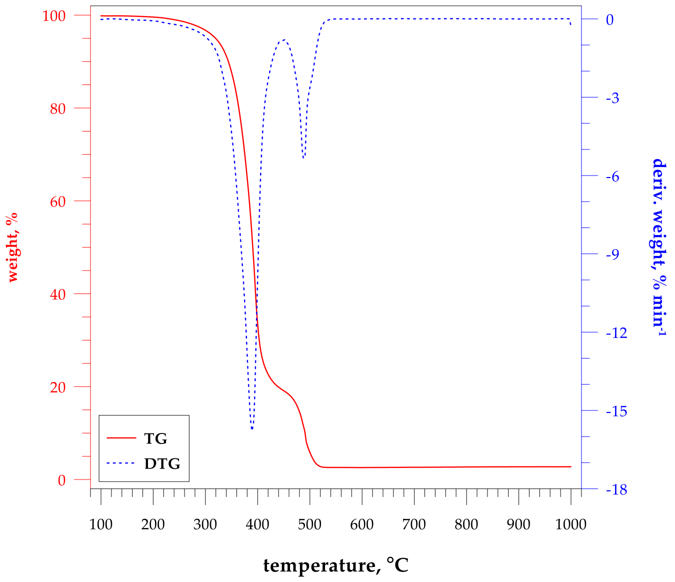

(d)

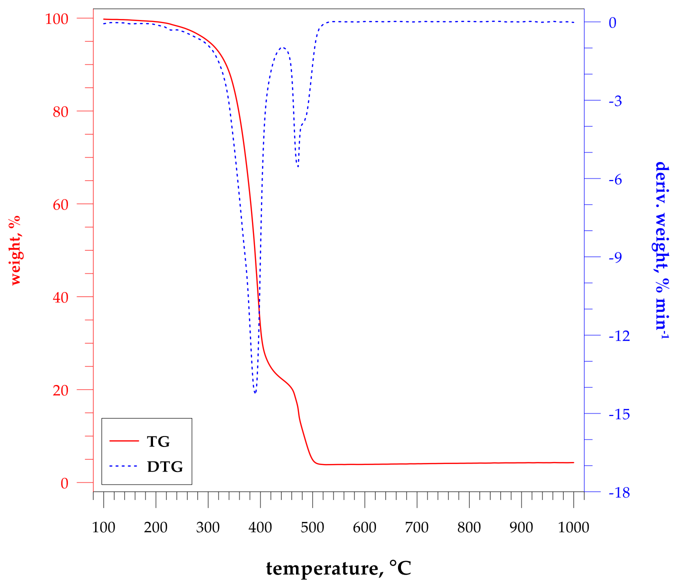

(e)

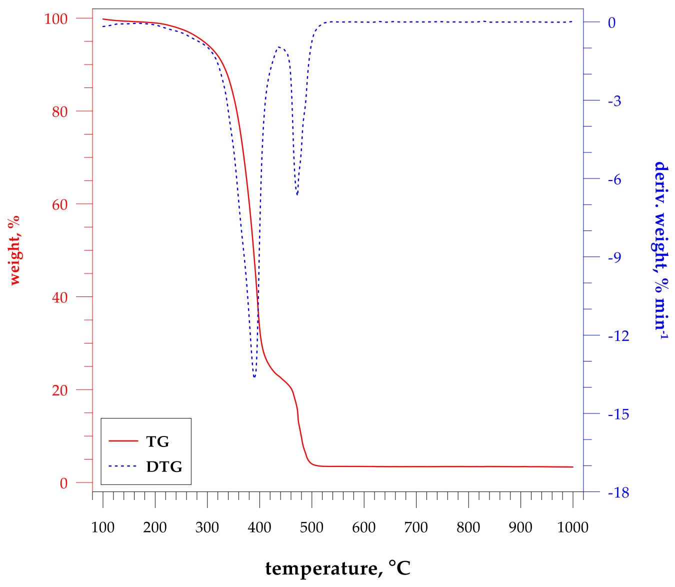

(f)

Figure S1. Thermal decomposition of the UPR/WSP composites. TG and DTG curves of (a) pure UPR; (b) walnut shell powder; (c) UPR+W2; (d) UPR+W5; (e) UPR+W7; (f) UPR+W10.

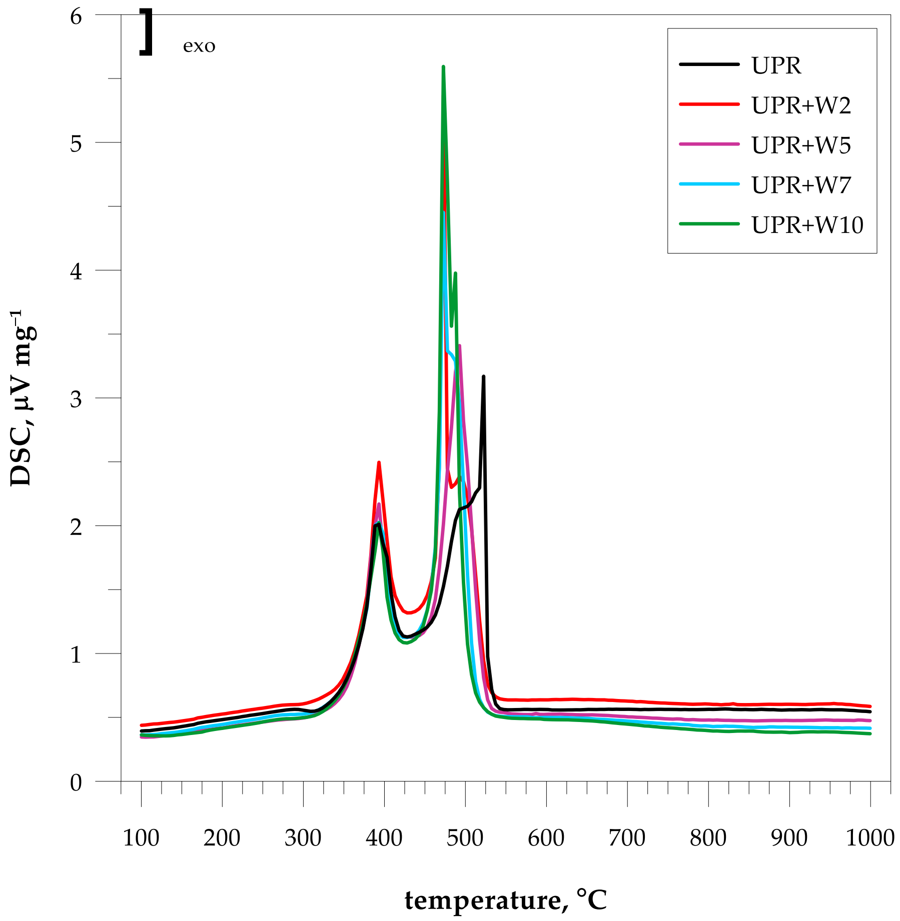

Figure S2. DSC curves of the UPR/WSP composites.
